# Sample-ready multiplex qPCR assay for detection of malaria

**DOI:** 10.1186/1475-2875-13-158

**Published:** 2014-04-25

**Authors:** Edwin Kamau, Saba Alemayehu, Karla C Feghali, Dennis W Juma, George M Blackstone, William R Marion, Peter Obare, Bernhards Ogutu, Christian F Ockenhouse

**Affiliations:** 1 Walter Reed Army Institute of Research, Military Malaria Research Program, Malaria Vaccine Branch, 503 Robert Grant Ave, Silver Spring, Maryland, USA; 2 Kenya Medical Research Institute (KEMRI), Global Emerging Infections Surveillance (GEIS) Program, United States Army Medical Research Unit-Kenya (USAMRU-K), Walter Reed Project, Kisumu, Kenya; 3 BioGX, 1500 1st Ave N # 34, Birmingham, AL, USA

## Abstract

**Background:**

Microscopy and antigen detecting rapid diagnostic tests are the diagnostic tests of choice in management of clinical malaria. However, due to their limitations, the need to utilize more sensitive methods such as real-time PCR (qPCR) is evident as more studies are now utilizing molecular methods in detection of malaria. Some of the challenges that continue to limit the widespread utilization of qPCR include lack of assay standardization, assay variability, risk of contamination, and the need for cold-chain. Lyophilization of molecular assays can overcome some of these limitations and potentially enable widespread qPCR utilization.

**Methods:**

A recently published multiplex malaria qPCR assay was lyophilized by freezing drying into Sample-Ready™ format (MMSR). MMSR assay contained all the required reagents for qPCR including primers and probes, requiring only the addition of water and sample to perform qPCR. The performance of the MMSR assay was compared to the non-freeze dried, “wet” assay. Stability studies were done by maintaining the MMSR assays at four different ambient temperatures of 4°C, room temperature (RT), 37°C and 42°C over a period of 42 days, tested at seven-day intervals. *Plasmodium falciparum* and *Plasmodium vivax* DNAs were used for analysis of the MMSR assay either as single or mixed parasites, at two different concentrations. The C_T_ values and the standard deviations (SD) were used in the analysis of the assay performance.

**Results:**

The limit of detection for the MMSR assay was 0.244 parasites/μL for Plasmodium spp. (PLU) and *P. falciparum* (FAL) assay targets compared to “wet” assay which was 0.39 and 3.13 parasites/μL for PLU and FAL assay targets, respectively. The MMSR assay performed with high efficiencies similar to those of the “wet” assay and was stable at 37°C for 42 days, with estimated shelf-life of 5 months. When used to analyse field clinical samples, MMSR assay performed with 100% sensitivity and specificity compared to the “wet” assay.

**Conclusion:**

The MMSR assay has the same robust performance characteristics as the “wet” assay and is highly stable. Availability of MMSR assay allows flexibility and provides an option in choosing assay for malaria diagnostics depending on the application, needs and budget.

## Background

Accurate and prompt diagnosis is essential for timely and appropriate treatment of malaria. The World Health Organization (WHO) guidelines for the treatment of malaria recommend parasitological confirmation of all suspected cases before treating [1]. Although microscopy remains the gold standard for malaria diagnosis [2,3], the use of malaria antigen detecting rapid diagnostic tests (mRDTs) as an alternative diagnostic method has increased over the years and provided an avenue through which access to parasite-based diagnosis, specifically in areas where good quality microscopy cannot be maintained, can be expanded [4]. However, both microscopy and mRDTs have many limitations. Notably, both methods have poor sensitivity and are highly variable [5-7]. Molecular assays that detect *Plasmodium*-specific nucleic acid sequences are increasingly being used in the detection and analysis of malaria to overcome limitations associated with microscopy and mRDTs. Assays such as real-time PCR (qPCR) are more sensitive and specific than microscopy and mRDTs [8,9], and have allowed for explicit identification and quantification of malaria parasites [8-13]. Most of the malaria qPCR assays target the multicopy 18S ribosomal RNA (rRNA) genes [14] with detection limits ranging from 0.002 to 30 parasites/μL [10,14].

Currently, there is lack of consensus on how to best perform and interpret qPCR assays [15]. Real-time PCRs can be performed in the background of different PCR master mixes, variable chemistry, reagents, and platforms, all which can affect the sensitivity and reproducibility of the assay. Furthermore, PCR requires specialized training and proper implementation of quality control measures, including need for a clean room or separate master mix preparation area. There is always the concern of false-positive results due to contamination or false-negative especially if proper internal controls are not in place. Real-time PCR also requires cold chain which can be expensive, highly variable and time consuming. Kamau *et al.*[16] recently described development of a multiplex qPCR assay for detection of *Plasmodium* genus target, and species specific *Plasmodium falciparum*, *Plasmodium vivax* targets as well as human RNaseP gene as an endogenous control. In addition to containing all the characteristics that makes qPCR an increasingly attractive diagnostic tool, the multiplex assay described was designed to be amenable to high throughput with drastically reduced costs.

This study describes further optimization of the recently published multiplex qPCR assay [16] which was lyophilized into a Sample-Ready™ format (BioGX, Birmingham, AL, USA). This format contains all the required components for qPCR; requiring only the addition of water and sample for qPCR analysis. Lyophilization of qPCR assays has many advantages and is important in mitigating some of the qPCR limitations. The Malaria Multiplex Sample-Ready™ (MMSR) assay was formulated with an optimized, highly sensitive chemistry with primers and probes for genus (*Plasmodium spp*. [PLU]) and species specific targets (*P. falciparum* [PAL] and *P. vivax* [VIV]) as well as human RNaseP gene. The performance characteristics of MMSR assay and stability studies are presented.

## Methods

### Samples

Samples used in this study were obtained from a blood collection protocol between 2010–2012 at the KEMRI/ Walter Reed Project, Kombewa District Hospital which is located in the Kombewa district, Kisumu County in western Kenya. The study was approved by the Ethical Review Committee of the Kenya Medical Research Institute (KEMRI), Nairobi, Kenya, and the Walter Reed Army Institute of Research (WRAIR) Institutional Review Board (IRB), Silver Spring, MD, USA. The study protocol numbers are KEMRI SSC NO. 2008 and WRAIR 1720. This study was conducted in accordance with GCP principles and instructions from the Department of Defense and the Department of the Army. All potential study subjects provided written informed consent before screening and enrollment and had to pass an assessment of understanding.

### *Plasmodium falciparum* reference reagent

The WHO international standard for *P. falciparum* DNA nucleic acid amplification technology (NAT) assays, obtained from the National Institute for Biological Standards and Control (NIBSC; Hertfordshire, UK) was used as the calibration reference reagent for the *Plasmodium spp*. and *P. falciparum* assays. The NIBSC standard consists of a freeze-dried preparation of whole blood collected by exchange transfusion from a patient infected with *P. falciparum*. Following NIBSC recommendations, this lyophilized material was suspended in 500 μL of sterile, nuclease-free water to a final concentration of 1×10^9^ IU/mL, which corresponds to a parasitaemia of 9.79 parasites/100 red blood cells [17,18]. The parasite density of the NIBSC standard after reconstitution was estimated to be 469,920 parasites/μL, based on the average red blood cell count (from uninfected donor) of 4.8×10^6^ RBC/μL. Unless otherwise indicated, fresh uninfected whole blood was used as a diluent to prepare serial dilutions. The uninfected whole blood was obtained from donors from Washington DC metropolitan area under WRAIR approved protocol. After reconstitution, genomic DNA was extracted with the EZ1 DNA blood kit on the EZ1 Advanced XL automated sample purification system (Qiagen, Valencia, CA, USA) as recommended by the manufacturer.

### Design and manufacturing of freeze dried Sample-Ready™ assays

The designing, development and testing of the “wet” qPCR assay have been previously described [10,16]. BioGX Inc, a molecular assay manufacturing company based in Birmingham, AL, USA was contracted to custom manufacture the MMSR assay using their proprietary procedures and formulations. BioGX Inc was supplied with all the primers, probes, and *Plasmodium* DNA for assay testing and optimization. They performed additional testing to optimize the MMSR assay using BioGX Inc master mix that is included in the final lyophilized Sample-Ready™ format. The MMSR assays were manufactured either as pellets or cakes in eight-well strip tubes or in 96-well plates, with each well containing all reagents for a 5-μL assay. The assays were sealed in water and lightproof pouches containing desiccant and either four-, six-well, eight-well strip tubes or one 96-well plate, which was either half or fully loaded. The MMSR assay was custom designed for use on an Applied Biosystems 7500 Fast Real-Time PCR System (Applied Biosystems, Foster City, CA, USA).

### Real-time PCR assays

Real-time qPCR amplifications and measurements for the “wet” assay were performed as previously described using QuantiTect Multiplex PCR Master Mix (Qiagen, Valencia, CA, USA) [16]. The qPCR amplification and measurements for MMSR assay were performed as follows: The assay was prepared by just adding 5 μL water to each test, and then adding 1 μL DNA (or water for non-template control (NTC)). The thermal profile used for the MMSR assay was as follows: 2 min at 95°C; 45 cycles of 10 sec at 95°C; 60 sec 59°C.

### Limit of detection (LoD) studies

The determination of the LoD began with dilution series. Reconstituted NIBSC standard or *P. vivax* sample were spiked into uninfected whole blood followed by a series of five-fold dilutions over five-fold range inclusive of the first dilution. This was followed by two-fold dilutions. DNA was extracted from each serially diluted sample using Qiagen EZ1 DSP DNA blood kit on EZ1 Advanced XL automated sample purification system. Extracted DNA samples were analysed using MMSR PCR assay with each sample tested in duplicate or triplicate. RNaseP assay was analysed in all the reactions. Reactions containing *P. falciparum* DNA (NIBSC standard) samples were analysed using *Plasmodium* spp. and *P. falciparum* assays whereas for the reactions containing *P. vivax* DNA sample, only *P. vivax* assay was used for analysis. The lowest concentration of DNA that yielded positive test results was considered the LoD.

### Stability studies

The stability of MMSR assay was estimated using accelerated aging techniques with elevated temperatures over a period of 42 days (estimated 1.5 months), analysed at intervals of seven days, referred to as time points. The ambient storage temperatures tested (test conditions) were 4°C (the storage conditions recommended by the manufacturer), room temperature (RT, set at 22°C and monitored frequently), 37°C and 42°C. For the 4°C ambient temperature, the assays were kept in the refrigerator with monitored temperature and for the RT, the assays were kept in clean area in the laboratory. For the elevated temperatures, two different incubators set at 37°C and 42°C were used for the entire period of study with continuous monitoring of the temperatures. For analysis, qPCR assays were performed every seven days. At the beginning of the study on D0, the number of assays required for testing at 4°C, RT, 37°C or 42°C on D7, D14, D21, D28, D35, and D42 were estimated. These MMSR assays were then kept at each ambient temperature conditions in zip-lock double bags with desiccants. On D7 and subsequent days, samples required for testing were retrieved and qPCR assay performed. The genomic DNA used in these experiments contained either *P. falciparum* only, *P. vivax* only or a mixture of both *P. falciparum* and *P. vivax* genomic DNAs. Two different concentrations of genomic DNA were used; high and low. The high concentration contained ~100 parasite/μL whereas the low contained ~20 parasite/μL. Each assay was performed in triplicate and C_T_ values were obtained. The standard deviation (SD) of the C_T_ values was used to assess the precision of the replicates as well as performance of the assays in the different conditions tested, including different ambient temperatures and period which the assay was stored. The longest duration that the thermostabilized PCR maintained its activity was calculated as described by Clark [19]:

Age of the thermostabilized PCR tubes = 1.5 months at 37°C or 42°C

Ambient temperature RT = 22°C

Q_10_ = 1.8

Acceleration factor at 37°C (based on 15°C temperature difference): (1.8)1.5 = 2 .41

Acceleration factor at 42°C (based on 20°C temperature difference): (1.8)2.0 = 3.24

Length of time at elevated temperature = 1.5 months

Estimation of shelf life:

Accelerated age = age × acceleration factor (at 37°C)

1.5 months × 2.41 = 3.615 months

Shelf life = accelerated age + actual age

3.615 + 1.5 = 5.115 months

Accelerated age = age × acceleration factor (at 42°C)

1.5 months × 3.24 = 4.86 months

Shelf life = accelerated age + actual age

8.1 + 2.5 = 6.36 months

## Results

### Performance characteristics of the MMSR assay

The initial performance characteristics of the MMSR assay were compared to the “wet” assay using NIBSC standard at three different concentrations as shown in Table 1. The assays were run in duplicate. The MMSR assay performed slightly better than “wet” assay by detecting the parasite DNA at lower C_T_ values. The sensitivity of the MMSR was compared to the “wet” assay by performing LoD experiments. The LoD for the MMSR assay was 0.244 parasites/μL for PLU and FAL targets compared to “wet” assay which was 0.39 and 3.13 parasites/μL for PLU and FAL targets, respectively. Lyophilization process improved the performance of the FAL assay, improving the LoD by ~ log from 3.13 parasites/μL to 0.244 parasites/μL. To evaluate the efficiency of MMSR assay, PLU, FAL and VIV plasmid DNAs were five-fold serially diluted five times and analysed in three replicates as previously described [16]. The MMSR assay performed with high efficiency and precision for all the targets similar to the “wet” assay as previously reported [16]. The PLU assay had efficiency of 99%, FAL assay 100% and VIV assay 99%, each with R^2^ values of 0.99 and SD replicates <0.167.

**Table 1:** Detection characteristics of MMSR assay in comparison to “wet” assay

	**MMSR assay**		**Wet assay**		**Parasite/μL**
	**C**_**T **_**Mean**	**C**_**T **_**SD**	**C**_**T **_**Mean**	**C**_**T **_**SD**	
**PLU assay**	18.55	0.013	19.78	0.191	78240
**FAL assay**	20.07	0.031	20.85	0.055	
**RNaseP assay**	24.13	0.083	30.09	0.081	
**PLU assay**	20.73	0.092	22.17	0.227	15648
**FAL assay**	22.23	0.054	23.62	0.338	
**RNaseP assay**	24.52	0.185	29.57	0.082	
**PLU assay**	23.58	0.029	24.89	0.616	3129
**FAL assay**	24.93	0.001	26.06	0.029	
**RNaseP assay**	25.37	0.055	29.43	0.492	

NIBSC standard DNA was five-fold serially diluted times. The performance of MMSR assay was compared to the “wet” assay. The performance of the MMSR assay was comparable to that of the “wet” assay for the three assay targets tested.

### MMSR stability studies

**Figure 1:**
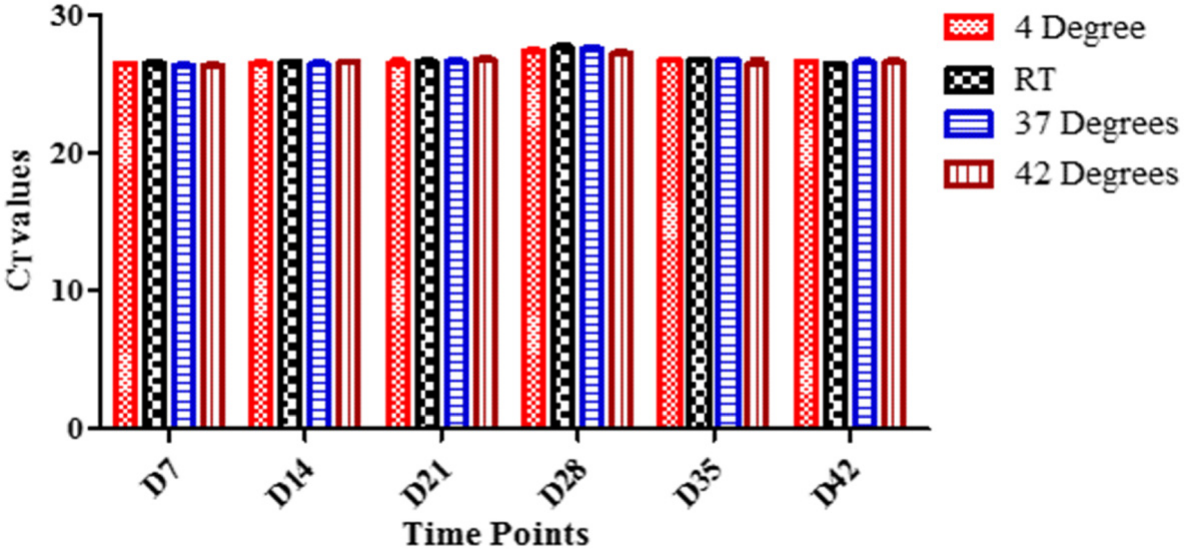
**Performance of PLU assay in different ambient temperatures.** Data showing C_T_ values obtained in all ambient temperatures tested on D7. The condition which the assay was stored did not impact the performance of the assay. Assay was analysed using DNA at high concentration.

**Figure 2:**
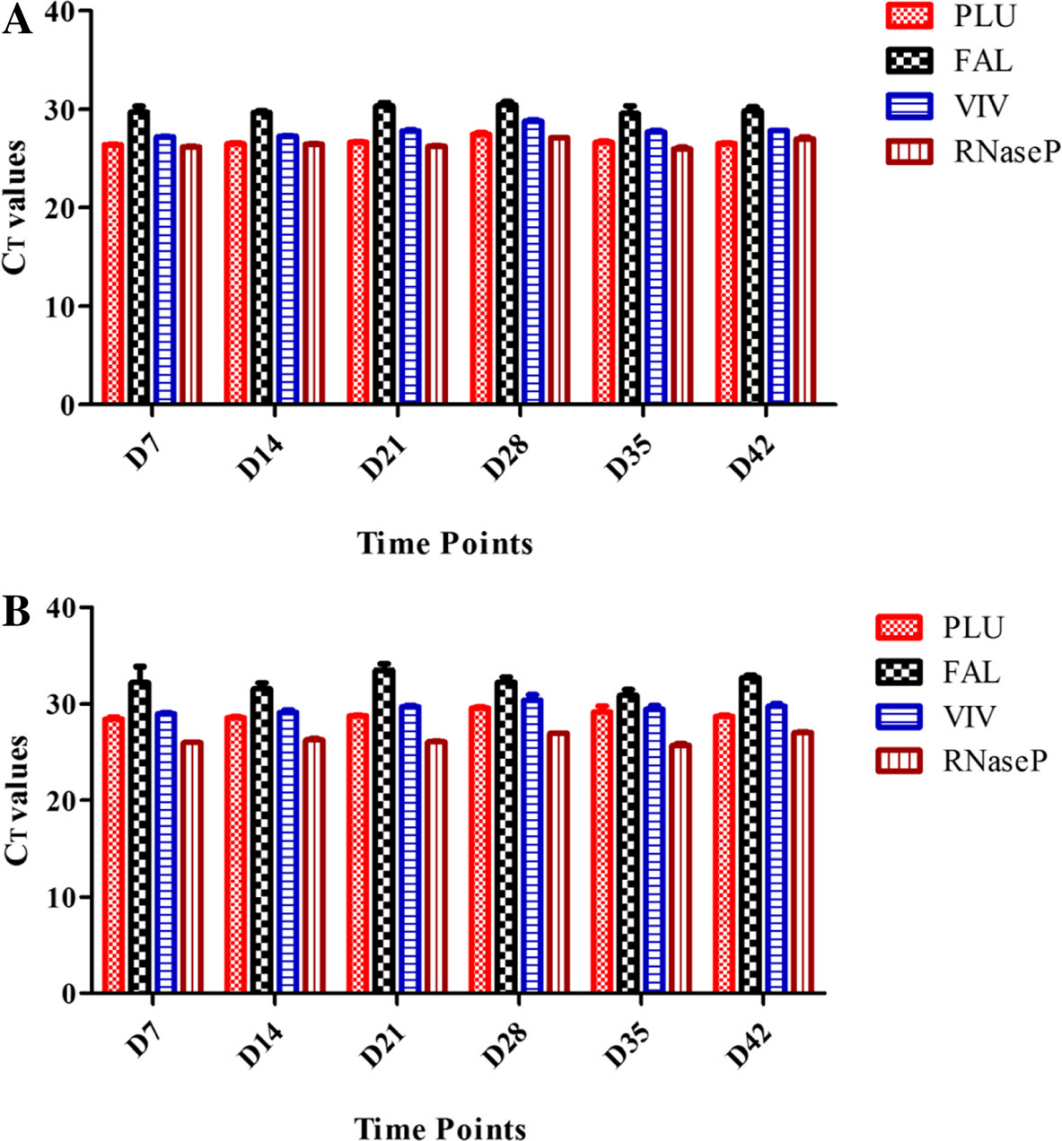
**Performance of individual assay targets over time.** Data showing the C_T_ mean ± SD for individual assay targets for all test conditions. Panel **A** shows data using DNA at high concentrations and panel **B** shows data using DNA at low concentration. For example, on D7, PLU assay is showing mean C_T_ values and the SD for each ambient temperature tested on that day. Low SD indicates different ambient temperatures which the assay was stored in did not impact the performance of the assay using either low or high DNA concentration. For FAL assay however, at low DNA concentration, the SD was larger than that of high DNA concentration indicating at low DNA concentration, the FAL assay performed with lower precision when the different ambient temperature being tested were compared.

The stability of MMSR assay was tested by comparing the performance of the assay at four different ambient temperatures over 42 days. The MMSR assay was manufactured in eight-well strip tubes or in 96-well plates with caps, and then sealed in a pouch, which contains desiccants. In addition, each pouch which contains the MMSR assays was put in zip-lock double bags with additional desiccants to minimize the possibility of moisture impacting the outcome of the study. Analyses of the MMSR assay using mixed DNA, which contained both *P. falciparum* and *P. vivax,* are presented here in detail. When the DNA at high concentration was analysed, the MMSR assay performed with high precision in all replicates and in all conditions tested (Additional file 1). Each individual assay targets, PLU, FAL, VIV and RNaseP were analysed by comparing the C_T_ values for each assay target, at each time point tested, for each condition. For example, on D7, PLU assay had C_T_ ± SD of 26.47 ± 0.056 at 4°C, 26.45 ± 0.18 at RT, 26.36 ± 0.04 at 37°C and 26.37 ± 0.078 at 42°C (Figure 1). These values had high precision with C_T_ mean ± SD 26.41 ± 0.06. This indicates on D7, the different ambient temperatures tested did not have an effect on the performance of the PLU target when used for analysis of DNA at high concentrations. Figure 2A shows the C_T_ mean ± SD for all individual assay targets for all test conditions at the different time points using DNA at high concentrations. As shown for D7, data obtained indicate that at each time point tested, each assay target performed equally well regardless of the ambient temperature. When the stability of MMSR assay was analysed using DNA at low concentrations, the performance of the assay indicated slight degradation at 37°C and 42°C on D21, D28 and D35 (Additional file 2). At 37°C, only one of the three replicates of VIV target was successfully amplified on D21 and D28. At 42°C, the VIV target failed to amplify on D28 and on D35, FAL and VIV assays failed. What is intriguing however, the four assay targets performed well on D42 at 42°C like they did on D0. The degradation is clearly indicated for specific target(s) since a mixture of DNA was loaded in the assay where some target(s) amplified while others failed to amplify in the same reaction. Figure 2B shows the C_T_ mean ± SD for all the individual assay targets for all test conditions at the different time points using DNA at low concentrations. Here, the SD values were larger compared to those obtained when DNA at higher concentrations was used (Additional file 1, Figure 2A), especially for FAL assay which performed with the least precision. MMSR assay performance was also analysed in all test conditions using single DNA of *P. falciparum* or *P. vivax*. The MMSR assay demonstrated high stability in all conditions tested when analysed with *P. vivax* DNA, both high and low concentrations. The degradation of the MMSR assay seen when mixed DNA at low concentration was used on D21, 28 and 35 at 37°C and 42°C were not evident when *P. vivax* DNA only was used. The MMSR assay was also stable when analysed with *P. falciparum* DNA only at low concentration. However, there was degradation of the PLU and FAL targets at 42°C from D14-D42.

### Comparison of MMSR assay to “wet” assay in analysis of field clinical samples

**Figure 3:**
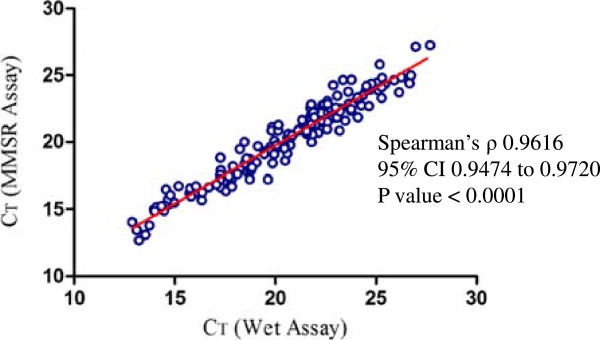
**MMSR assay *versus *wet assay in analysis of field clinical samples.** Spearman’s analysis between analysis of clinical samples using MMSR and wet assay revealed there was a statistically significant correlation between the two assays.

To further compare the performance of MMSR assay to the “wet” assay, 180 field clinical samples were analysed using both assay formulations. The “wet” assay was used as the reference method which the performance of the MMSR assay was compared against. The sensitivity of MMSR assay was calculated as [(number of true positives)/(number of true positives + number of false negatives)], and specificity was calculated as the [(number of true negatives)/(number of true negatives + number of false positives)] as previously described [8]. The assay performed with 100% sensitivity and specificity. To further compare the performance of the two assay formulations using field clinical samples, Spearman’s analysis was performed using C_T_ values obtained (Figure 3). The statistical analysis revealed that the pairing was highly effective with p <0.0001. The mean C_T_ for MMSR assay was 20.67 (95% CI 20.13-21.21) and for the wet assay was 20.29 (95% CI 19.82-20.77), further revealing the performance characteristics of these two assay formulations was highly comparable.

## Discussion

This study describes creation of a lyophilized, MMSR assay for detection of *Plasmodium* DNA targets and an endogenous control. The initial characterization compared the performance of the MMSR assay to that of the “wet” assay which was recently published [16]. Some of the characteristics that were initially assessed include assay efficiency, precision and sensitivity. The efficiency of a PCR reaction is critical because it is a measure of the overall performance of the assay whereas precision can be used to monitor the accuracy of template and reagent pipetting, homogeneity of template, and instrument performance. The MMSR assay performed with high efficiency, precision and was slightly more sensitive than the “wet” assay. The improved sensitivity seen with lyophilized assay can be explained by the fact that when manufacturing these assays, the Taq DNA polymerase used is glycerol free. This allows for higher concentration of Taq DNA polymerase to be used per reaction in the lyophilized assay than in the “wet” assay. Furthermore, the way the lyophilized assay is formulated allows for adjustments of other components in the lyophilization master mix (by addition or subtraction) such as various salts and additives that cannot knowledgeably be changed in commercial off-the-shelf master mix (such as QuantiTect) used in the “wet” assay. These performance characteristics of the MMSR assay demonstrated confidence that conversion of the “wet” assay into lyophilized assay did not compromise the performance of the malaria multiplex assay.

The ready-to-use lyophilized qPCR assays mitigates some of the qPCR limitations. They simplify the workflow, reduce variability, reduce risk of contamination, and remove the need for cold-chain. Stability of lyophilized PCR assays and the removal of the need for cold-chain is critical as qPCR continues to become an important diagnostic tool in resource constraint environment. It also removes the challenges and cost associated with shipping and storage of qPCR reagents. This study focused on testing the stability of the MMSR assay in different ambient temperature conditions. Previous studies have demonstrated stability of lyophilized PCR assays that last beyond six months at temperatures ranging between 20 and 24°C, and three months at 37°C [20-23]. The MMSR assay was stable in all test conditions when high DNA concentration was used for analysis over the entire period which the assay was tested. However, at low DNA concentration, some of the assay targets were degraded when analysed using mixed DNA at elevated temperatures. The RNaseP assay remained stable in all test conditions. Using the Q_10_ method, the stability of medical devices is tested by storing the device at elevated temperature and monitoring stability overtime [19]. The longest duration that the thermostabilized PCR maintains its activity is calculated to determine the shelf-life of the device [19,21]. In this study, the thermostability of MMSR assay was tested for 42 days, which is approximately 1.5 months. At 37°C, the MMSR assay was stable at all the test conditions analysed using *P. falciparum*, *P. vivax* or mixed DNA, both at low and high DNA concentrations. The only exception was when only one of the three replicate amplified on D21 and on D28, when mixed DNA was used for analysis. The thermostability of the MMSR assay was calculated at 37°C for 1.5 months, which the shelf life for MMSR assay at ambient temperature RT of 22°C was estimated to be 5 months when mathematically correlated with its stability at 37°C. The stability of MMSR assay is important because it clearly demonstrates that the MMSR assay can be transported and stored without the need for cold-chain, a useful and critical attribute especially for distributing this test to remote or distance regions without the concern of degradation of the reagents or decreased reliability of the test. Currently, the military malaria programme has footprint in the USA, Southeast Asia, South America and Africa. The MMSR assay is currently being used to support studies in some of these locations. The stability of the assay is strategic because it ensures assay reproducibility regardless of location which the assay is performed. It also removes transportation challenges and costs associated with the need for cold-chain.

To further validate the utility of MMSR assay in field settings, samples collected from field studies were tested at USAMRU-K laboratories in Kenya. The MMSR assay used was over 18 months old, stored at 4°C and transported from the USA to Kenya without cold-chain. The data obtained demonstrated that the MMSR assay performed the same as the “wet” assay, validating the utility of the MMSR assay in field settings. This further demonstrates that MMSR assay can easily be developed for personal qPCR instruments or point-of-care instruments without concerns of compromising the assay sensitivity.

BioGX Inc, the manufacturer of MMSR assay, has extensive experience in designing and manufacturing of lyophilized Sample-Ready™ assays. They provide custom molecular assay design, development, automation, and manufacturing services and offers qPCR tests across diverse applications. For example, in recent news (posted on BioGX Inc’ website), the US Food and Drug Administration released a protocol to identify ruminant DNA in pig-derived crude heparin utilizing BioGX Inc's real-time PCR-based Ruminant and Porcine test. In the development of the MMSR assay, two prototypes of the Sample-Ready™ assays were developed; pellets and cakes. The pellets form a smooth round shaped product whereas cakes form a white substance dispensed at the bottom of the tube. The pellets are easy to work with because they settle at the bottom of the tube. Cakes however tend to flake and can be challenging to work with because of the amount of static developed, which can lead to loss of the material. BioGX Inc performed several studies to optimize the cake and prescribed procedures to control statics. They recommended using the cake formulation because it would provide a more sensitive and stable product.

In addition to BioGX Inc, there are other companies, such as GE Life Sciences (Pittsburgh, PA, USA) and Cepheid (Sunnyvale, CA, USA) that can custom manufacture lyophilized PCR assays. It is also possible to custom-make and optimize lyophilized PCR assays in the laboratory as well. However, this can be a long, tedious and complex process. For example, in an attempt to lyophilize an internal positive control RNA to help ensure the accuracy of the detection of avian influenza virus RNA by reverse transcription (RT)-PCR and real-time RT-PCR, Das *et al.*[20] were forced to lyophilize without some of the enzymes because of stability concerns. Klatser *et al.* describes a complex procedure for developing of a freeze-dried, lyophilized PCR assay [22]. In 2010, Cheung *et al*. [24] attempted to custom-make lyophilized assay for detection of influenza. However, the lyophilized reagents and the assay condition needed further optimization to improve the sensitivity and amplification efficiency. However, when a PubMed search was done in January 2014, there was no indication such optimization had been published more than three years later. Having a company custom-manufacture lyophilized assay(s) is likely to result in a more optimized, reproducible product, much more than attempting to custom-make lyophilized assay in a laboratory due to complexity of the procedure. However, the lyophilization process is likely to increase the cost of qPCR assay which is one of the main concerns of molecular assays. At the current rate, “wet” assay cost less than one dollar per reaction whereas lyophilized assay is more than ten dollars per reaction, increasing the cost of qPCR per reaction by more than ten-fold. It is therefore critical that the benefits of lyophilized molecular assays are assessed in the context of all the benefits gained with lyophilization and the cost savings that come along with it, such as reduced cost in transporting of reagents and no need for cold-chain.

## Conclusion

A stable, lyophilized multiplex assay for detection of *Plasmodium*, *P. falciparum* and *P. vivax* that is highly robust and has the same performance characteristics as the “wet” assay has been developed. Although the lyophilization process may increase the cost of qPCR, both assays can be adapted for analysis of samples depending on the situation and the prevailing circumstances. Most important, since both formulations have the same performance characteristics, assays performed using either of the formulations can be compared. Both lyophilized and “wet” assays are routinely used for qPCR analysis at the WRAIR and subordinate commands. Both formulations have been used in controlled human malaria infection (CHMI) trials. MMSR assay is especially critical in CHMI studies where precision, reproducibility and sensitivity of the qPCR assay are important and cost of individual assays is not a limiting factor since fewer samples are analysed compared to field studies. Availability of alternative formulations of qPCR assay with the same performance characteristics, each with unique advantages, offers the flexibility of choosing the most convenient diagnostic tool from the molecular diagnostics toolkit.

## Competing interests

The authors declare that they have no competing interests.

## Authors’ contributions

EK and CFO conceived the project idea. EK and SA designed the experiments. GMB and WRM optimized the freeze-dried MMSR assay and manufactured the prototype. BO was the PI of the blood collection protocol. PO provided samples from the blood collection protocol. SA, KCF, EK, DWJ performed the experiments. SA and EK analysed the data. EK and SA wrote the manuscript and EK, SA, DWJ, KCF, GMB, PO, BO and CFO reviewed the manuscript. All authors approved the final version of the manuscript.

## Supplementary Material

Additional file 1 **Data showing the C**_**T **_**values (mean ± SD) obtained for each individual assay targets for each test condition at the seven different time points using DNA at high concentration.** Click here for file

Addition file 2 **Data showing the C**_**T **_**values (mean ± SD) obtained for each individual assay targets for each test condition at the 7 different time points using DNA at low concentration.** Click here for file
