# Supplementary material for: Sample-ready multiplex qPCR assay for detection of malaria
Source: Malar J. 2014 Apr 25;13:158. doi: 10.1186/1475-2875-13-158 (PMC4026594; doi:10.1186/1475-2875-13-158)
Supplement: Additional file 1 — Data showing the C T values (mean ± SD) obtained for each individual assay targets for each test condition at the seven different time points using DNA at high concentration. [file 1475-2875-13-158-S1.docx]

|  | D0 | D7 | D14 | D21 | D28 | D35 | D42 |
| --- | --- | --- | --- | --- | --- | --- | --- |
| PLU | 26.49±0.05 | 26.47±0.06 | 26.50±0.05 | 26.52±0.12 | 27.40±0.06 | 26.70±0.01 | 26.61±0.02 |
| FAL | 29.41±0.27 | 29.28±0.27 | 29.54±0.27 | 30.24±0.15 | 30.34±0.10 | 29.13±0.29 | 29.80±0.12 |
| VIV | 27.28±0.16 | 27.21±0.13 | 27.35±0.18 | 27.75±0.12 | 28.83±0.11 | 27.83±0.06 | 27.91±0.05 |
| RNaseP | 26.30±0.06 | 26.17±0.03 | 26.43±0.09 | 26.29±0.13 | 27.18±0.04 | 26.09±0.09 | 27.21±0.05 |
|  |  |  |  |  |  |  |  |
| PLU |  | 26.45±0.18 | 26.58±0.04 | 26.64±0.09 | 27.64±0.21 | 26.75±0.08 | 26.44±0.04 |
| FAL |  | 30.22±0.30 | 29.91±0.35 | 30.21±0.26 | 30.28±0.28 | 29.04±0.13 | 29.86±0.33 |
| VIV |  | 27.25±0.13 | 27.33±0.04 | 27.77±0.16 | 28.96±0.18 | 27.85±0.12 | 27.83±0.03 |
| RNaseP |  | 26.26±0.13 | 26.46±0.10 | 26.29±0.02 | 27.09±0.03 | 26.14±0.07 | 27.07±0.07 |
|  |  |  |  |  |  |  |  |
| PLU |  | 26.36±0.04 | 26.38±0.17 | 26.64±0.12 | 27.53±0.17 | 26.70±0.12 | 26.54±0.15 |
| FAL |  | 29.18±0.02 | 29.68±0.26 | 30.11±0.05 | 30.19±0.21 | 29.41±0.10 | 29.34±0.01 |
| VIV |  | 27.13±0.08 | 27.23±0.21 | 27.91±0.10 | 28.84±0.10 | 27.58±0.13 | 27.81±0.21 |
| RNaseP |  | 26.24±0.16 | 26.33±0.06 | 26.23±0.05 | 27.10±0.08 | 25.94±0.05 | 26.85±0.13 |
|  |  |  |  |  |  |  |  |
| PLU |  | 26.37±0.08 | 26.58±0.04 | 26.77±0.11 | 27.21±0.16 | 26.47±0.25 | 26.54±0.15 |
| FAL |  | 30.24±0.19 | 29.54±0.10 | 30.85±0.30 | 30.95±0.19 | 30.70±0.25 | 30.35±0.33 |
| VIV |  | 27.24±0.19 | 27.29±0.25 | 27.87±0.09 | 28.60±0.15 | 27.52±0.23 | 27.81±0.21 |
| RNaseP |  | 26.10±0.03 | 26.54±0.17 | 26.14±0.07 | 27.10±0.23 | 25.80±0.16 | 26.85±0.13 |

Additional file 1 **Data showing the CT values (mean ± SD) obtained for each individual assay targets for each test condition at the seven different time points using DNA at high concentration**

RT

4 °C

37 °C

42 °C
