# Supplementary material for: Sample-ready multiplex qPCR assay for detection of malaria
Source: Malar J. 2014 Apr 25;13:158. doi: 10.1186/1475-2875-13-158 (PMC4026594; doi:10.1186/1475-2875-13-158)
Supplement: Addition file 2 — Data showing the C T values (mean ± SD) obtained for each individual assay targets for each test condition at the 7 different time points using DNA at low concentration. [file 1475-2875-13-158-S2.docx]

Addition file 2 **Data showing the CT values (mean ± SD) obtained for each individual assay targets for each test condition at the 7 different time points using DNA at low concentration**

|  | D0 | D7 | D14 | D21 | D28 | D35 | D42 |
| --- | --- | --- | --- | --- | --- | --- | --- |
| PLU | 28.62±0.13 | 28.30±0.07 | 28.63±0.26 | 28.75±0.12 | 29.75±0.15 | 28.83±0.61 | 28.90±0.21 |
| FAL | 30.94±0.20 | 30.66±0.30 | 32.00±0.88 | 33.66±0.29 | 31.56±0.15 | 31.51±0.74 | 31.39±0.00 |
| VIV | 29.30±0.29 | 28.85±0.03 | 29.52±0.40 | 29.71±0.50 | 30.94±0.17 | 29.38±0.47 | 30.16±0.33 |
| RNaseP | 26.02±0.07 | 26.02±0.02 | 26.48±0.21 | 26.10±0.08 | 27.00±0.04 | 25.95±0.12 | 27.12±0.15 |
|  |  |  |  |  |  |  |  |
| PLU |  | 28.18±0.18 | 28.64±0.15 | 28.88±0.06 | 29.62±0.53 | 28.94±0.08 | 28.59±0.09 |
| FAL |  | 31.38±0.62 | 31.58±0.30 | 33.58±0.83 | 32.26±0.11 | 30.95±0.07 | 32.36±0.19 |
| VIV |  | 29.14±0.04 | 29.10±0.31 | 29.94±0.12 | 30.54 | 29.87±0.58 | 29.55±0.12 |
| RNaseP |  | 26.06±0.05 | 26.25±0.08 | 26.14±0.09 | 27.03±0.04 | 25.79±0.20 | 27.12±0.05 |
|  |  |  |  |  |  |  |  |
| PLU |  | 28.60±0.12 | 28.65±0.26 | 28.75±0.28 | 29.36±0.51 | 28.78±0.36 | 28.76±0.23 |
| FAL |  | 32.60±0.09 | 30.67±0.34 | 32.67±0.82 | 32.67±0.05 | 30.29±0.13 | 33.37±0.41 |
| VIV |  | 28.87±0.12 | 29.03±0.23 | 29.54 | 29.82 | 29.18±0.01 | 29.79±0.32 |
| RNaseP |  | 25.94±0.07 | 26.23±0.07 | 26.09±0.09 | 26.93±0.03 | 25.60±0.10 | 27.01±0.05 |
|  |  |  |  |  |  |  |  |
| PLU |  | 28.68±0.26 | 28.36±0.04 | 28.73±0.16 | 29.56±0.05 | 30.13±0.43 | 28.79±0.12 |
| FAL |  | 34.37±0.81 | 32.06±0.30 | 34.15±2.82 | 32.65±0.69 |  | 33.91±0.84 |
| VIV |  | 29.09±0.19 | 28.91±0.20 | 29.66±0.17 |  |  | 29.67±0.33 |
| RNaseP |  | 25.99±0.04 | 26.14±0.24 | 25.93±0.08 | 26.92±0.10 | 25.45±0.03 | 26.90±0.71 |

4 °C

RT

37 °C

42 °C
